# Dietary Bioactives in Alzheimer’s Disease: A Critical Appraisal of Clinical Trials and Future Nutritional Strategies

**DOI:** 10.3390/nu18060907

**Published:** 2026-03-12

**Authors:** Ankita Kumari, Xin-An Zeng

**Affiliations:** 1 School of Food Science and Engineering, South China University of Technology, Guangzhou 510640, China; anki93.ag@gmail.com; 2 Guangdong Key Laboratory of Food Intelligent Manufacturing, Foshan University, Foshan 528225, China; 3 School of Food Science and Engineering, Foshan University, Foshan 528225, China; 4 Overseas Expertise Introduction Centre for Discipline Innovation of Food Nutrition and Human Health (111 Centre), Guangzhou 510640, China

**Keywords:** Alzheimer’s disease (AD), dietary bioactives (DBs), cognitive decline, mild cognitive impairment (MCI), clinical trial, precision nutrition, nutraceutical

## Abstract

**Background:** Alzheimer’s disease (AD) remains a major public health challenge. Observational associations between dietary patterns and reduced dementia risk have prompted investigations of dietary bioactives (DBs) as cognitive nutraceuticals. **Methods:** This critical narrative review examines interventional trials for nine prominent DBs relevant to AD: docosahexaenoic acid (DHA), curcumin, resveratrol, epigallocatechin gallate (EGCG), nicotinamide riboside (NR), tricaprilin, vitamin E (α-tocopherol), cannabinoids, and NIC5-15 (D-pinitol). Trials were identified through ClinicalTrials.gov (search date: December 2024) and supplemented by PubMed searches for published results. Data were extracted on trial phase, design, cognitive/functional endpoints, biomarker outcomes, and development status. Findings are synthesized qualitatively; no formal meta-analysis or risk of bias assessment was conducted. **Results:** None of the nine bioactives demonstrated consistent cognitive efficacy in AD. Phase III trials of DHA, curcumin, and tricaprilin did not meet primary cognitive endpoints. Resveratrol reduced CSF Aβ40 without cognitive benefit. Cannabinoids improved behavioral symptoms but showed no measurable cognitive effects. High-dose vitamin E slowed functional decline, while cognition remained unchanged. In contrast, trials in preclinical or at-risk populations reported preliminary cognitive signals for EGCG and biomarker engagement for NR, suggesting potential for early intervention. **Conclusions:** Current clinical evidence does not support high-dose DBs supplementation as an effective treatment for AD. Predominantly negative late-phase findings highlight limitations, with potential contributors including limited bioavailability, late intervention, insufficient target engagement, and biological heterogeneity. Future research may benefit from early biomarker-defined populations, optimized formulations, multi-nutrient or dietary approaches, and precision nutrition strategies considering genetic risk and baseline nutrient status. DBs may be better positioned for prevention or early-stage intervention rather than late-stage therapy.

## 1. Introduction

The prevalence of Alzheimer’s disease (AD) is projected to escalate in line with rising life expectancies across various nations. A recent analysis estimates that by 2050, the global population aged 40 and older living with dementia will rise to about 153 million, up from 57 million in 2019. This increase is mainly due to population growth and aging. All regions will be affected, with North Africa and the Middle East expecting a 367% rise in cases, while eastern sub-Saharan Africa anticipates a 357% increase [1]. This data highlights a significant concern about the disease’s pathophysiology and preventive strategies. In parallel, epidemiological evidence has consistently linked specific dietary patterns, such as the Mediterranean and MIND diets, to a reduced risk of cognitive decline and AD [2]. These patterns are rich in bioactive food components, suggesting a critical role for nutrition in brain health.

AD is a cause of progressive deterioration of specific neurons in certain brain areas, leading to cytoskeletal changes, neuronal dysfunction, subsequent degeneration, and brain atrophy [3,4]. As the disorder progresses, individuals with AD experience a decline in episodic memory, language, and executive functioning, as well as behavioral deficits. The pathophysiology of AD is thought to begin with the accumulation of amyloid-β (Aβ) in the brain’s cortical regions approximately 10–30 years before the onset of dementia [5,6]. The aggregation of Aβ peptides, neurofibrillary tangles (NFTs), and inflammation are hypothesized to be the underlying causes of AD pathogenesis. These changes progress to reduced brain volume and are responsible for the memory loss associated with AD [7]. Cognitive impairment in patients with AD presents with varying degrees of severity. The preclinical stage may present as a subjective decline in mental abilities, even if objective cognitive testing shows no impairment [8]. Mild cognitive impairment (MCI) is the initial symptomatic stage, where one or more cognitive domains are mildly impaired, but daily functioning remains mostly intact [9]. In contrast, dementia is described as a cognitive impairment severe enough to affect an individual’s independence and daily functioning. However, the duration that individuals with amyloid pathology spend in each stage is still uncertain [10].

**Figure 1:**
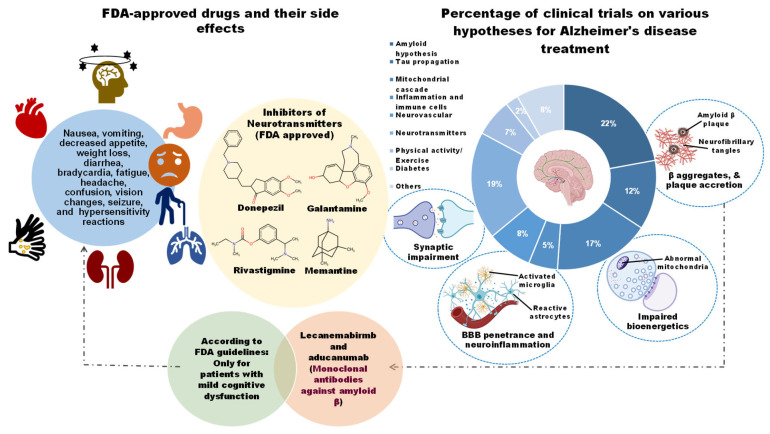
An overview of FDA-approved drugs, their associated side effects, and the percentage of clinical trials covering the various hypotheses tested as of 2019. Overall, the amyloid hypothesis was tested the most with 22.3% of trials, the second-most tested was the neurotransmitter hypothesis with 19.0%, while other hypotheses, such as tau proliferation were around 12.7%, mitochondrial signaling at 17%, neurovascular system at 7.9%, physical activity 6.6%, inflammation 4.6%, viral related tests 0.5% and other unclassified tests 8.4%. Furthermore, two FDA-approved anti-amyloid monoclonal antibodies, lecanemab and aducanumab, are breakthrough agents for early AD that have many side effects in patients. Similarly, the four FDA-approved drugs donepezil, galantamine, rivastigmine, and memantine are blockers of neurotransmitters that produce in patients similar adverse events as those two monoclonal antibodies, including nausea, vomiting, decreased appetite, weight loss, etc.

This chronic disease has become one of the most burdensome and costly ailments due to the lack of a proper therapeutic strategy. Over the past two decades, enormous clinical trials have been performed to obtain AD-modifying therapies. These trials have tested a wide range of hypotheses, including the amyloid (22.3%), neurotransmitters (19%), Tau propagation (12.2%), mitochondrial cascade (17%), neurovascular (7.9%), and inflammatory hypothesis (4.6%), among others (Figure 1). Still, no potential drug has been developed to ease disease phenotypes [11]. Although clinically approved medications such as cholinesterase inhibitors (e.g., donepezil, galantamine, and rivastigmine) and an *N*-methyl-D-aspartic acid (NMDA) receptor antagonist (e.g., memantine) significantly improve the symptoms, they do not reduce AD progression [12]. Abnormal accretions of Aβ caused by dysregulated proteolytic processing of APP (Amyloid precursor protein) by major secretases (α/β/γ-secretases) are pathogenic to AD progression [13]. However, inhibitors of BACE1 (β-secretase) (e.g., AN-1792, atabecestat, bapineuzumab, solanezumab, gantenerumab, verubecestat, lanabecestat, aducanumab, and elenbecestat (E2609), as well as selective immunotherapy against Aβ, do not show noticeable efficacy in ameliorating cognitive function in AD patients [14]. Only a limited number of pharmacological agents aimed at Aβ and tau proteins remain in clinical trials. While recent advances with monoclonal antibodies targeting amyloid have marked a turning point, their modest efficacy, high cost, and safety concerns underscore the critical need for complementary and preventive strategies.

In this landscape, dietary bioactives (DBs) have garnered immense interest in nutritional neuroscience. Throughout this review, “dietary bioactives” refer to food-based compounds that, beyond their basic nutritional effects, exert measurable biological effects on physiological processes relevant to health and disease. This definition aligns with the Office of Dietary Supplements (NIH, Bethesda, MD, USA) framework, which characterizes bioactives as food or supplement constituents, excluding essential nutrients that influence cellular activities, metabolic pathways, or disease risk [15]. In the Alzheimer’s context, DBs of interest include polyphenols (curcumin, resveratrol, EGCG), omega-3 fatty acids (DHA), pleiotropic vitamins (vitamin E), metabolic modulators (nicotinamide riboside, tricaprilin), and neuroactive plant compounds (cannabinoids, D-pinitol). Their appeal lies in pleiotropic mechanisms that simultaneously target multiple AD pathways, including amyloid aggregation, oxidative stress, and neuroinflammation, while maintaining favorable safety profiles [16]. Preclinical studies have consistently demonstrated these multi-target effects, and specific DBs such as nicotinamide riboside and tricaprilin further address the profound metabolic and bioenergetic deficits characteristic of the AD brain [17].

The therapeutic relevance of DBs is underscored by their historical and continued contributions to modern medicine. Many compounds first identified in edible and medicinal plants have served as precursors for clinically approved drugs, demonstrating a direct translational pathway from botanical source to physiological modulator. Notable examples include galantamine, a cholinesterase inhibitor sourced from daffodil (*Amaryllidaceae*), and aspirin, derived from willow bark salicylates [18]. This is not an isolated trend; an analysis of new chemical entities approved between 1981 and 2020 reveals that a majority originated from natural substances, predominantly higher plants, with only 36% being entirely synthetic [19,20]. This statistic highlights plants as a vast reservoir of bioactive scaffolds, a principle that positions dietary bioactives as promising candidates for nutritional intervention in complex diseases like AD.

**Figure 2:**
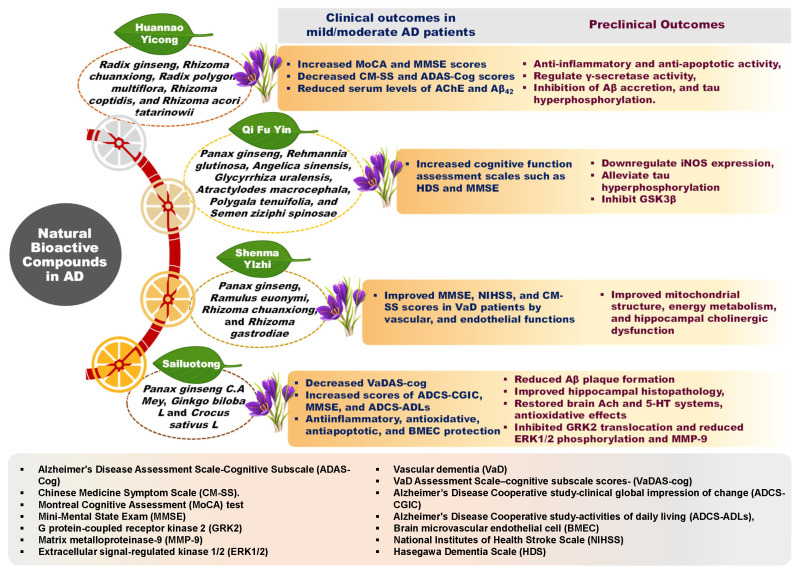
*Panax ginseng*-enriched natural Chinese medicinal formulations and their DB efficiency in preclinical and clinical trials. These DBs have significantly improved the different assessment scores related to cognitive functions and daily life activities of mild/moderate AD patients. In preclinical models, these DBs have shown anti-inflammatory, antioxidant, anti-apoptotic actions, etc. The abbreviations for various scores are shown in the box below.

Furthermore, many DBs under investigation exhibit a significant likelihood of prior use in traditional medicinal systems, which often leveraged whole plants or complex herbal formulations. These traditional approaches posit that the combined effects of multiple bioactive compounds within a plant matrix can lead to enhanced outcomes through synergistic interactions [21]. For instance, as illustrated in Figure 2, various ginseng-enriched traditional Chinese medicinal formulations have been evaluated for their synergistic potential in managing AD symptoms [22]. A salient case of retrospective recognition is the anti-cancer agent Taxol (paclitaxel), derived from the Pacific yew (*Taxus brevifolia*), whose ethnomedicinal use was identified after its discovery via modern screening [23,24]. Hence, integrating insights from traditional food and medicinal practices with contemporary nutritional science may offer innovative, multi-targeted avenues for dietary intervention in AD.

According to the AD drug development pipeline 2024, several of these dietary bioactives are currently under clinical investigation as repurposed agents, with preliminary outcomes warranting deeper analysis [25]. However, translating compelling preclinical findings into consistent, tangible clinical benefits has proven highly challenging. The clinical trial history for these compounds reflects a heterogeneous landscape, characterised by unmet primary endpoints, occasional subgroup signals, and subsequent shifts in research focus. While select agents, such as vitamin E, have demonstrated specific clinical value, many others have been abandoned following negative trials or have had their investigation shifted entirely.

Therefore, this review first explores the multifactorial pathobiology of AD to contextualize the theoretical rationale for multi-target DBs. It then thoroughly synthesizes and critically appraises the clinical trial evidence from registries such as ClinicalTrials.gov for nine prominent DBs. We aim to move beyond preclinical promise and provide a clear-eyed, evidence-based evaluation of the human clinical data. By distilling key lessons from past failures and strategic shifts, we outline a rational and promising path forward for nutraceuticals and dietary interventions in AD. Specifically, this review aims to: (i) critically evaluate reported efficacy across cognitive, functional, and biomarker outcomes; (ii) examine plausible factors underlying inconsistent or negative clinical findings; and (iii) propose evidence-informed directions for future nutritional and early-intervention strategies in AD.

## 2. Methods

### 2.1. Compound Selection Criteria

The nine dietary bioactives examined in this review, docosahexaenoic acid (DHA), curcumin, resveratrol, epigallocatechin gallate (EGCG), nicotinamide riboside (NR), tricaprilin, vitamin E (α-tocopherol), cannabinoids, and NIC5-15 (D-pinitol), were selected based on a conceptual selection framework informed by the existing literature, including:

**Preclinical Rationale:** Evidence of neuroprotective mechanisms relevant to AD pathophysiology, including modulation of amyloid aggregation, tau pathology, neuroinflammation, oxidative stress, mitochondrial function, or synaptic plasticity.

**Clinical Trial Representation:** Presence of completed or ongoing interventional trials registered on ClinicalTrials.gov investigating the compound in AD, mild cognitive impairment (MCI), or preclinical AD populations, with sufficient available data to permit critical evaluation.

**Scientific and Translational Relevance:** Compounds demonstrating substantial research interest and translational potential within the nutritional neuroscience and neurodegeneration literature.

Compounds were excluded if they lacked AD-specific clinical trial data, were tested only in combination formulations without clear isolation of the active component, or had no registered interventional trials on ClinicalTrials.gov as of the search date. These criteria were applied to ensure both biological plausibility and clinical relevance of included compounds.

### 2.2. Search Strategy and Data Sources

This review used a structured search approach to identify interventional clinical trials investigating dietary bioactives (DBs) for Alzheimer’s disease (AD) and related neurocognitive disorders. As this study is intended as a **critical narrative synthesis**, the search strategy was designed to provide a transparent overview of relevant clinical trials rather than to follow formal systematic review or meta-analysis methodology. Searches were conducted to capture the breadth of available clinical trial evidence while prioritizing studies most relevant to the scope of this review.

**Registry:** ClinicalTrials.gov (maintained by the U.S. National Library of Medicine, NIH).

**Search Date:** The primary search was conducted in December 2024.

**Search Fields:** The following fields were queried: Condition/Disease, Intervention/Treatment, Study Type, Phase, Status, and Age Group.

Search Terms (Condition):

- “Alzheimer disease” OR “Alzheimer’s Disease” OR “Dementia”.
- “Mild Cognitive Impairment” OR “MCI”.
- “Prodromal Alzheimer” OR “Preclinical Alzheimer”.

**Search Terms (Intervention):** For each of the nine predefined compounds, the following search syntax was applied:

- (“docosahexaenoic acid” OR “DHA”) AND (“Alzheimer*” OR “dementia” OR “MCI” OR “cognition”).
- (“curcumin” OR “turmeric”) AND (“Alzheimer*” OR “dementia” OR “MCI” OR “cognition”).
- (“resveratrol”) AND (“Alzheimer*” OR “dementia” OR “MCI” OR “cognition”).
- (“epigallocatechin gallate” OR “EGCG”) AND (“Alzheimer*” OR “dementia” OR “MCI” OR “cognition”).
- (“nicotinamide riboside” OR “NR”) AND (“Alzheimer*” OR “dementia” OR “MCI” OR “cognition”).
- (“tricaprilin” OR “AC-1202”) AND (“Alzheimer*” OR “dementia” OR “MCI” OR “cognition”).
- (“vitamin E” OR “alpha-tocopherol”) AND (“Alzheimer*” OR “dementia” OR “MCI” OR “cognition”).
- (“cannabinoids” OR “THC” OR “cannabidiol”) AND (“Alzheimer*” OR “dementia” OR “MCI” OR “cognition”).
- (“NIC5-15” OR “D-pinitol”) AND (“Alzheimer*” OR “dementia” OR “MCI” OR “cognition”).

Truncation operators were applied where appropriate to capture variations in terminology (e.g., Alzheimer, Alzheimer’s).

Filters Applied:

- **Study Type:** Interventional Studies (Clinical Trials).
- **Phase:** All phases (I, II, III, IV, Not Applicable).
- **Status:** Completed, Terminated, Withdrawn.
- **Age Group:** Adult (18–64), Older Adult (65+).
- **Study Results:** All studies (both with and without posted results).

Definition of “Completed”: In accordance with the ClinicalTrials.gov data dictionary, a trial is classified as “Completed” when it has regularly concluded and participants are no longer being examined or treated (i.e., the last participant’s last visit has occurred). This status is distinct from “Terminated” (halted prematurely and will not resume) and “Withdrawn” (stopped prior to enrollment of the first participant). Trials with “Terminated” or “Withdrawn” status were deliberately included to avoid positive publication bias and to capture evidence from discontinued or failed interventions.

Supplementary Literature Search: To address the limitation that many completed trials lack posted results on ClinicalTrials.gov, we conducted a complementary search of PubMed (MEDLINE) for peer-reviewed publications associated with each registered trial. Search terms included the trial registry identifier (NCT number) and combinations of compound name and condition. Conference abstracts and preprints were not systematically included but were referenced selectively when registry data were incomplete, and no full publication was available.

### 2.3. Study Selection Framework

Trials identified through the search strategy were reviewed and considered for inclusion based on the following guiding inclusion and exclusion considerations:

Inclusion Criteria:

- **Study Design:** Interventional trials (defined as studies in which participants are assigned to receive one or more interventions to evaluate effects on health-related outcomes).
- **Population:** Participants diagnosed with AD (any stage), MCI due to AD, prodromal AD, or preclinical AD.
- **Intervention:** Trials evaluating one or more of the nine predefined dietary bioactives as the primary intervention, administered orally or via other routes as a single agent or as the clearly isolated active component.
- **Minimum Duration:** No minimum trial duration was imposed as an inclusion criterion. However, trials with intervention periods shorter than 4 weeks were considered unlikely to detect meaningful cognitive change and were interpreted cautiously in the narrative synthesis.
- **Required Endpoints:** Trials were required to report at least one cognitive, functional, or biomarker outcome. Trials registered without any such outcome measures were excluded.

Exclusion Criteria:

- **Population:** Trials focused on non-AD dementias (e.g., vascular dementia, frontotemporal dementia, Parkinson’s disease dementia, Lewy body dementia); trials where MCI was attributable to non-AD etiologies (e.g., Parkinson’s disease, traumatic brain injury, psychiatric disorders, HIV-associated neurocognitive disorders).
- **Intervention:** Non-pharmacologic interventions (e.g., exercise, cognitive training, lifestyle coaching, caregiver support, dance therapy, music therapy) unless the bioactive compound was the primary and isolated intervention; trials of multivitamins, proprietary combination products, or medical foods where the specific bioactive could not be isolated and evaluated independently.
- **Study Design:** Observational studies, case reports, case series, preclinical studies, narrative reviews, systematic reviews, and meta-analyses.

### 2.4. Data Extraction and Synthesis

For each trial included in the narrative synthesis, the following data were compiled by the authors from registry records and associated publications, with discrepancies resolved through discussion:

Trial Characteristics:

- NCT identifier, trial title, phase, status (Completed, Terminated, Withdrawn), enrollment, study design (randomization, blinding, parallel/crossover), intervention duration, dosing regimen, comparator/placebo.

Participant Characteristics:

- Diagnostic criteria (AD, MCI, prodromal, preclinical), sample size, age, sex, APOE genotype stratification (if reported), baseline cognitive status.

Outcome Measures and Endpoints:

- **Cognitive endpoints:** Alzheimer’s Disease Assessment Scale-Cognitive Subscale (ADAS-Cog), Mini-Mental State Examination (MMSE), Clinical Dementia Rating-Sum of Boxes (CDR-SB), domain-specific neuropsychological test batteries.
- **Functional endpoints:** Alzheimer’s Disease Cooperative Study-Activities of Daily Living (ADCS-ADL), Disability Assessment for Dementia (DAD), Montreal Cognitive Assessment (MoCA).
- **Biomarker endpoints:** Cerebrospinal fluid (CSF) Aβ42, CSF total tau and phosphorylated tau (p-tau), plasma Aβ42/Aβ40 ratio, plasma p-tau181, plasma neurofilament light chain (NfL), neuroimaging (amyloid PET, FDG-PET, MRI volumetry), blood-based metabolic markers (e.g., NAD+ levels, ketone bodies, inflammatory cytokines).
- **Safety and tolerability:** Adverse events, withdrawals due to adverse events.

Handling of Heterogeneity and Missing Data:

Given the substantial variability in outcome measures, follow-up durations, dosing regimens, and participant populations across trials, no quantitative synthesis (meta-analysis) was performed. Instead, a qualitative narrative synthesis organized by compound was conducted. For each compound, we described the direction, magnitude, and statistical significance of reported effects on cognitive, functional, and biomarker outcomes.

Where multiple trial arms existed, we prioritized comparisons between active intervention and placebo, focusing on the highest dose or most bioavailable formulation evaluated. Missing data were not imputed; trials with unreported, incomplete, or non-disaggregated outcome data were noted as such in the narrative synthesis.

### 2.5. Quality Assessment Statement

A formal risk-of-bias assessment (e.g., RoB 2.0) and GRADE evaluation were not performed, consistent with the objectives of a critical narrative review. This review, therefore, does not aim to provide a formal grading of evidence certainty. The aim was to evaluate the direction, consistency, and translational relevance of clinical trial evidence rather than to quantify methodological certainty. Accordingly, no claims regarding the internal validity, risk of bias, or certainty of evidence are made. Readers should interpret the synthesized evidence with this methodological limitation in mind. Descriptive design features (randomization, blinding, placebo control, sample size, attrition) are reported where available to provide context for interpreting individual trial findings.

### 2.6. Limitations of the Search and Synthesis Approach

This review relied primarily on trial records from ClinicalTrials.gov supplemented by peer-reviewed publications identified via PubMed. This approach carries inherent limitations:

1. **Incomplete registry data:** Many completed trials lack posted results on ClinicalTrials.gov, requiring manual searches for published outcomes. Despite cross-referencing, we cannot ensure complete capture of all trial outcomes.
2. **Outdated or unsynchronized records:** Registry records may not be updated following trial completion or publication, leading to potential discrepancies between registered protocols and final published outcomes.
3. **Underreporting of terminated trials:** Trials terminated for futility or safety concerns are frequently underreported in the peer-reviewed literature. We addressed this by intentionally including “Terminated” and “Withdrawn” status trials and cross-referencing registry data with conference abstracts and press releases where available. Nevertheless, publication bias cannot be fully excluded.
4. **Language and database restriction:** Searches were limited to ClinicalTrials.gov and PubMed; trials registered exclusively in non-U.S. registries (e.g., EU Clinical Trials Register, ISRCTN, ChiCTR) or published in languages other than English may not be represented.

These limitations are taken into account in the interpretation of our findings, and the evidence base for several compounds remains fragmented. We have aimed for transparency rather than comprehensiveness, consistent with the critical narrative review format. While every effort was made to enhance transparency and minimize selection bias, this review should be interpreted as an evidence-mapping and interpretative narrative synthesis, rather than a formal systematic review or meta-analysis.

## 3. The Complex Pathobiology of AD: Implications for Multi-Target Nutritional Strategies

The complex, multifactorial nature of AD pathophysiology presents a fundamental challenge for single-target pharmacotherapies but provides a compelling theoretical rationale for interventions with multi-target capabilities, such as dietary DBs. This section outlines the key interconnected pathological pathways in AD: amyloid, tau, neuroinflammation, mitochondrial dysfunction, and synaptic failure. It then highlights how their interplay underscores the potential utility of pleiotropic DBs.

**Figure 3:**
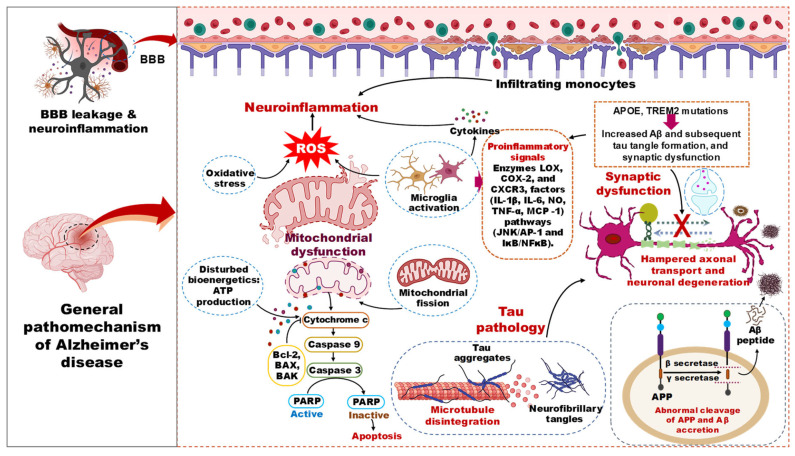
The multifactorial etiology of AD represents many pathological events with biochemical and cellular phases. Amyloidogenic processing of APP stimulated by genetic factors/aging contributes to forming aggregates of monomeric Aβ into oligomers, then into fibrils, which finally become part of extracellular amyloid plaques. Furthermore, these small aggregates cause reactive oxygen species (ROS) production and mitochondrial dysfunction, followed by tau aggregation and neurofibrillary tangles. The inflammatory pathway involves several incentives, including infiltrating monocytes because of BBB breakage. Meanwhile, activation of astrocytes and microglia leads to the secretion of proinflammatory cytokines and ROS, a subsequent cause of neuronal oxidative stress and mitochondrial dysfunction. Recruitment of phagocytic microglia into compromised neurons/neurites and release of harmful cytokines during clearance of extracellular debris increases bystander neurotoxicity, affecting adjacent neurons and senile plaque widening. Increased ROS during cellular respiration causes a decrease in mitochondrial membrane potential and ATP production by negatively affecting mitochondrial energy stores, disturbing energy metabolism, and compromising motility and mitophagy. This event ultimately increases caspase activity, which initiates apoptosis. Thus, increased mitochondrial ROS formation, compromised mitochondrial function, and apoptosis (caspase-3-dependent proteolytic cleavage of its substrate PARP) are associated with excessive mitochondrial fission and the loss of mitochondrial integrity. Moreover, mutations in APOE and TREM2 are specifically related to progressive disintegration in neuronal networks due to greater susceptibility to Aβ accretion and tau tangles formation, which hinder axonal transport and cause synaptic dysfunction and neuronal degeneration. These events damage the energetic efficiency of the brain.

The pathophysiology of AD involves failures in multiple cellular, molecular, and cortical circuitry organizations, leading to cognitive impairment and synaptic dysfunction. The disease is characterized by two types of lesions, namely an obvious lesion (microscopic neurofibrillary tangles, Aβ plaques, activated glial cells, and enlarged endosomes) and a more insidious lesion (loss of synaptic homeostasis and neuronal network integrity) [26,27]. The biochemical phase of AD is driven by the aggregation of Aβ peptides, which precipitates the misfolding and propagation of tau proteins, culminating in the formation of neurotoxic oligomers of both Aβ and tau (Figure 3). The age-related decline in the proteostasis network and dysfunction of autophagic mechanisms are implicated as contributing factors to the accrual of these protein aggregates [28,29]. The discrete positions of Aβ and tau pathology development in the brain suggest the contribution of two independent biochemical pathologies [30]. Once these two pathological events reach threshold levels, their interplay can exacerbate the disease state by producing deleterious effects on multiple cellular events [31]. Hence, increased proteopathic stress of Aβ and tau, along with aging, are the risk factors for sporadic AD.

### 3.1. Amyloid Beta Pathology in AD

To slow the progression of AD, therapeutic strategies that aim to prevent the formation of Aβ, reduce its soluble levels in the brain, prevent its aggregation into plaques, and break down existing Aβ plaques have been employed. However, during the past two decades, this approach has come under criticism due to negative trial results and limited efficacy, with some questioning whether Aβ is the right or appropriate target for treating AD. Despite these concerns, anti-Aβ approaches are the most advanced in development and represent the first class of disease-modifying treatments approved by the FDA [32]. Several monoclonal antibodies designed to target various forms of soluble and insoluble Aβ species have advanced to phase 3 trials [33,34,35,36,37]. However, these antibodies targeting soluble Aβ species, such as solanezumab and crenezumab, did not effectively clear amyloid plaques from the brain or demonstrate significant changes in cognition compared to placebo in phase 3 trials [33,34]. Additionally, current anti-amyloid therapies have raised safety concerns, as three deaths were reported in patients treated with lecanemab who developed intracerebral hemorrhage after the primary trial [38]. To mitigate this adverse response, a potential future strategy could involve targeting other components of amyloid plaques, such as APOE (Apolipoprotein E), to address both CAA (Cerebral amyloid angiopathy) and parenchymal plaque pathology. The positive impact of anti-amyloid treatments on slowing cognitive decline in symptomatic AD patients supports the need to test these therapies in the preclinical stage of the disease. However, conducting trials in this stage is challenging due to the long time required to observe the transition to clinical AD. Recent secondary prevention trials in autosomal dominant AD using anti-amyloid agents were not successful in slowing cognitive decline, possibly due to the low number of participants who experienced decline during the studies [39]. There are still questions and concerns about the best trial design for assessing preventive treatments in healthy, asymptomatic individuals, especially with drugs that can cause amyloid-related imaging abnormalities [40]. Despite these challenges, the recent approval of lecanemab and upcoming Aβ-monoclonal antibodies will likely spur further research in AD prevention trials, including developing new approaches to clearing amyloids. Moreover, enzyme BACE1, as the initiator of Aβ production, seemed like a promising solution to prevent AD. However, clinical trials of BACE inhibitors (e.g., atabecestat, LY3202626, verubecestat, elenbecestat, umibecestat, and lanabecestat) [41] fell short of expectations and were linked to mild, temporary cognitive decline [42,43]. However, a closer look at the data has revealed that low doses of BACE inhibition can still reduce Aβ production, much like the protective mutation that reduces AD risk. For asymptomatic patients with no significant plaque buildup or related symptoms, this could be a viable solution to slow the clinical progression of the disease [44]. Future interventions targeting other pathologies, such as tau, neuroinflammation, and cerebrovascular disease, along with early intervention in the AD continuum, are expected to enhance the efficacy of these treatments. The limited success of highly selective anti-amyloid agents contrasts with the potential of certain dietary DBs like resveratrol and EGCG, which may indirectly modulate amyloid pathology through anti-aggregation, anti-inflammatory, and enhancement of clearance pathways, representing a broader, multi-modal approach.

### 3.2. Tau Pathology in AD

Besides Aβ pathology, AD is characterized by the accumulation of tau protein aggregates in the brain, leading to multiple tauopathies. The spread of these aggregates has been linked to the progression of the disease, making immunotherapy a viable treatment option. Interestingly, tau lesions are more strongly correlated with the degree of dementia than Aβ plaques, suggesting that their clearance may be more effective in treating AD. Currently, there are two active vaccines (AADvac1 and ACI35) and six antibodies (LY3303560, RO7105705, BMS-986168, C2N8E12, JNJ-63733657, and UCB0107) in active clinical development for tau immunotherapy. However, most of these treatments are still in the early stages of development and target late-stage pre-clinical AD [45]. To better address the various stages of AD, combination therapy using anti-Aβ and anti-tau approaches is being tested using the ATN disease staging framework (A: amyloid, T: phosphorylated tau, and N: neurodegeneration) [46]. The hope is that combining these treatments may provide additive or even synergistic benefits. Recent studies suggest that local replication of tau drives accumulation and neurofibrillary tangle formation, instead of spreading between brain regions [47]. Therefore, it is necessary to test whether anti-tau immunization strategies can prevent the pathological spreading of tau pathology in AD. However, whether a pathological form of ‘free’ extracellular tau drives tau spreading is still unclear, so the effectiveness of anti-tau antibody treatments is uncertain. Currently, there are no approved drugs that directly target tau. However, several anti-tau drugs are being developed, with 31% in preclinical stages, 16% in discovery stages, 23% actively undergoing clinical trials (phases I, II, and III), and 28% of drug programs are inactive, meaning they have not been updated recently. Unfortunately, there are very few drug candidates in later clinical phases, with only four therapeutics in phase III and 16 in phase II [48]. Thus, tau-targeting therapeutics are being developed to reduce the assemblage of toxic forms of tau within neurons in preclinical or early AD. However, none have shown clear clinical efficacy to date [49]. One possible reason for these failures is the inability of anti-tau therapies to engage with soluble forms of tau within neurons [50,51]. Therefore, future research must focus on developing reliable translational animal models and selective compounds to target tau-specific epitopes, neurotoxic aggregates, and post-translational modifications in next-generation anti-tau therapies. The challenge of engaging intracellular tau with antibodies highlights a potential niche for small-molecule DBs, such as formulations of curcumin or specific flavonoids, which can cross cell membranes and may influence tau phosphorylation and aggregation via kinase modulation or direct interaction.

### 3.3. Neuroinflammation in AD Pathology

Disease-modifying therapies targeting neuroinflammatory molecular pathways face challenges due to the dynamic nature of immune reactions in individual cells and the intricate coordination among various cell types that drive both innate and adaptive immune responses [52]. For example, defective TREM2 (Triggering receptor expressed on myeloid cells 2) impairs the microglial function of Aβ plaques, worsening tissue damage, whereas its overexpression reduces the severity of AD pathology. The discovery of TREM2 loss-of-function variants as a risk factor for late-onset AD supports the use of TREM2 agonists as a therapeutic approach for AD [53,54]. In preclinical mouse models, TREM2 has been shown to activate microglia, leading to their clustering around amyloid plaques. This helps reduce neuritic dystrophy and the spread of amyloid-associated tau [55], while also contributing to a reactive microglial state that causes neuronal loss [56,57]. Moreover, TREM2 agonism has produced both aggravating and diminishing effects on AD-related pathologies in different animal models [58,59]. While activating TREM2 may effectively treat early stages of AD characterized by amyloid accumulation, its efficacy during the symptomatic stage, when tau pathology is present, remains uncertain. Therapeutic strategies to promote microglial activation in response to amyloid pathology may become complicated by pro-inflammatory responses. Future therapeutic agents may need to facilitate microglial efferocytosis, an anti-inflammatory process that clears apoptotic cells and promotes tissue repair [60]. A recent report indicated a strategy to enhance efferocytosis with chimeric antigen receptors [61], suggesting a potential approach to target the elimination of AD-related pathologies. Additionally, APOE influences microglial responses in both TREM2-dependent and TREM2-independent ways in AD pathologies [62], primarily through its expression in cerebral astrocytes, reactive microglia, and stressed neurons, which coordinate innate immune responses in AD pathology [63]. Recent findings indicate that the immune system, with a particular emphasis on innate immunity, plays a crucial role in the pathophysiology of AD. Targeting neuroinflammation could, therefore, serve as a therapeutic approach to complement recently approved immunotherapy using anti-Aβ antibodies. Several investigational drugs targeting immunomodulators, inflammatory signaling pathways, and CNS resident cells are being evaluated clinically at varying stages of AD [64]. Many agents, such as kinase inhibitors (P38 MAPK, ERK/NF-kB, and Janus kinase), pro-inflammatory cytokine inhibitors (lenalidomide and emtricitabine), a TNF inhibitor (XPro1595), an IL-1 inhibitor (canakinumab), and modulators of microglia and astrocyte activation (including AL002, TB006, edicotinib, sargramostim, daratumumab, and pepinemab), are under clinical trials. In addition, eicosanoid inhibitors (Salsalate, ALZT-OP1, and montelukast) are also being studied [64]. Each approach has its advantages and disadvantages; however, targeting CNS-resident microglia and astrocytes, considered major instigators of AD, has attracted significant attention. The complex, dual role of neuroinflammation makes it a difficult drug target but an ideal candidate for modulation by DBs with known immunomodulatory properties (e.g., curcumin, epigallocatechin gallate, omega-3 fatty acids), which can subtly temper excessive inflammation without causing broad immunosuppression.

### 3.4. Mitochondrial Dysfunction in AD

Research on AD has focused on cholinergic, Aβ, and tau dysfunction, with recent findings showing connections between mitochondrial dysfunction and classical AD targets. Mitochondrial dysfunction can lead to increased production of Aβ and hyperphosphorylation of Tau proteins, contributing to neurodegeneration [65]. In AD, mitochondria exhibit abnormal morphology due to imbalanced fission and fusion reactions, resulting in swollen mitochondria with distorted structure and abnormal distribution near the cell nucleus [66,67]. The primary evidence of mitochondrial dysfunction underlying AD progression comes from the brain’s abnormal energy hypometabolism observed in AD patients [68], which occurs due to altered microvasculature leading to reduced blood and oxygen supply to neurons [69]. Impaired energy metabolism causes the formation of abnormal protein molecules and a significant rise in the number of ROS in AD subjects, resulting in substantial increases in cerebral lipid peroxidation and protein and DNA/RNA oxidation, leading to neuronal death [70]. Currently, disease-modifying agents such as Metformin (Phase III), Tricaprilin (Phase III), Insulin + Empagliflozin (Phase II), Choline (Phase II), Edaravone (Phase II), and Nicotinamide Riboside (Phase I) targeting mitochondrial bioenergetics and oxidative stress are under clinical trial [25]. Existing therapies address mitochondrial dysfunction, bioenergetics, antioxidants, anti-apoptotic, glucose metabolism, and mitochondrial biogenesis and resistance [71]. However, limited pharmacotherapies are subject to clinical trials due to low efficacy. One such agent is Dimebon, which opens the mitochondrial permeability transition pores and has shown promising effects on memory and cognition in AD patients in a phase II trial [72]. At the same time, the results were not supported in the Phase III trial, possibly due to the heterogeneous population of neuropathology [73]. Some challenges, such as enabling drugs to penetrate the BBB (Blood–brain barrier) to reach mitochondria and triggering the action of drugs only inside mitochondria to minimize potential side effects, are critical in designing mitochondrial therapies [74]. Thus, the development of therapies targeting mitochondria is still in the early stages, and no breakthrough has been achieved yet. This direct link between energy failure and pathology is a key target for nutritional metabokinesis. Nicotinamide riboside (to boost NAD+) and tricaprilin (to provide ketone bodies) are prime examples of DBs in clinical trials specifically designed to address this bioenergetic deficit.

### 3.5. Synaptic Dysfunction in AD

In the CNS, synapses are crucial in transmitting electrical or chemical signals among neurons and are tightly regulated concerning their location and timing. Synaptic loss is closely associated with cognitive decline in AD patients, indicating that the integrity of synapses may have a causal role in the development of AD [75]. Aβ oligomers, particularly type 2, and soluble forms of tau proteins are implicated in damaging synapses; however, how these proteins impair synaptic function remains questionable [75,76]. Synaptic dysfunction in AD encompasses alterations in the structure and function of presynaptic terminals, dendrites, postsynaptic elements, and the synaptic cleft. Additionally, impairments in mitochondrial energy supply and the clearance of synapses by microglia may also contribute to synaptic dysfunction [77]. The early drug discovery efforts in AD focused on improving the efficiency of remaining synapses in AD brains [78]. Some drugs like cholinesterase inhibitors and the NMDA receptor antagonist are approved for AD treatment, but their effectiveness is limited [79]. This might be due to their inability to address ongoing synaptic loss. An alternative strategy involves promoting the brain’s natural compensatory mechanisms to counter synaptic loss in AD. The brain can compensate for synapse loss, which may explain the delayed onset of memory deficits in AD [80]. Boosting the brain’s natural compensatory capacity could be an effective approach for treating synapse loss in the early stages of AD [81]. Novel delivery methods, like encapsulated cell biodelivery, are being developed to address these challenges in humans [82]. Several disease-modifying small molecules are under phase III trials, such as Blarcamesine (σ-1 and M2 receptor agonist) and Fosgonimeton (Hepatocyte growth factor), to enhance neuronal survival and synaptic plasticity and reduce aberrant neuronal hyperactivity and tau-induced neurodegeneration in AD. While other drugs such as 50561 (RAC1 inhibitor), ABBV-552 (SV2A modulator), Dalzanemdor (NMDA receptor blocker), EX039 (DAO inhibitor), MW150 (p38 α MAPK kinase inhibitor) are in phase II trials and Levetiracetam (SV2A modulator), Simufilam (Filamin A conformation stabilizer) in both phase II/III for investigating their potential in synaptic plasticity and neuroprotection [25]. It is anticipated that positive findings from one or more of these trials will inspire further research. Supporting synaptic resilience and plasticity through nutritional means is an active area of exploration. Omega-3 fatty acids (e.g., DHA), flavonoids, and specific herbal extracts are being studied for their ability to support membrane fluidity, synaptic signaling, and neurotrophic factor expression, offering a preventive, pro-resilience strategy.

In summary, AD is propelled by a self-reinforcing network of amyloidosis, tauopathy, neuroinflammation, bioenergetic failure, and synaptic disintegration. The repeated shortcomings of highly selective pharmacological agents against single nodes in this network highlight its resilience to mono-target approaches. This pathobiological complexity, however, creates a compelling rationale for DBs investigation. Their inherent pleiotropy, the ability to gently modulate several of these interconnected pathways simultaneously, positions them as uniquely suited candidates for preventive and early-intervention strategies. This mechanistic foundation directly informs the critical analysis of their clinical trial evidence that follows.

## 4. Clinical Trial Evidence for Dietary Bioactives (DBs)

**Figure 4:**
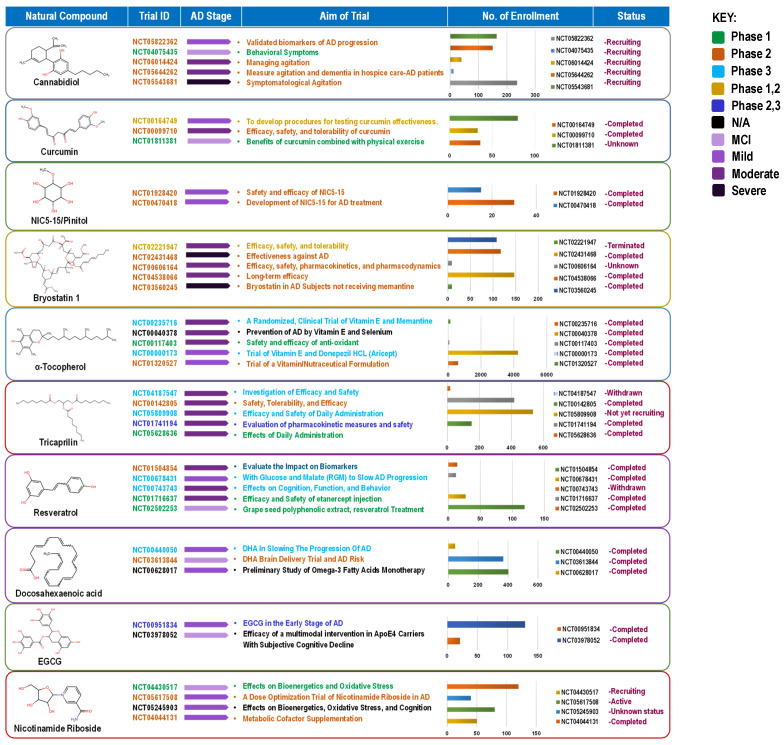
Numerous clinical trials have been conducted to study the neuroprotective efficiency of DBs as a primary or supplementary therapy. The figure illustrates the DBs alongside their respective clinical trial numbers, study objectives, participant numbers, and trial status. The color scale indicates the stage of the clinical trials (i.e., different colors of clinical trial IDs denote their phase) and whether the study involved AD patients with mild to severe symptoms, i.e., AD stage.

The compounds reviewed here, cannabinoids, curcumin, vitamin E, DHA, resveratrol, EGCG, and NR, are not only investigated as DBs but are also integral components of various whole foods and dietary patterns. Their investigation in high-dose, isolated supplemental form represents a specific, pharmaceutical-like approach within the broader spectrum of nutritional intervention. This section critically analyzes this supplemental paradigm, while acknowledging that the ultimate translation of this evidence may lie in its integration within complex diets and food matrices for long-term, low-intensity preventive strategies (Figure 4).

### 4.1. Cannabinoids: Efficacy for Behavioral Symptoms, Not Cognition

Cannabinoids (e.g., tetrahydrocannabinol) from the Cannabis plant have recently been considered for treating neuropsychiatric symptoms in AD patients. This interest stems from cannabinoids’ ability to interact with the endocannabinoid system, which plays a critical role in modulating neuroinflammatory processes and influencing mood and behavior. To date, cannabis and cannabis-derived compounds have not been approved by the FDA to treat or manage Alzheimer’s, and only a few clinical trials to evaluate the use of THC (Tetrahydrocannabinol, e.g., dronabinol and nabilone) or CBM (cannabinoid-based medicines) have been completed or are ongoing. A trial with 21 participants (mean age 85, with 77% female) indicated decreased agitation and improved relaxation and sleep with CBM [83]. In a study with 22 patients (mean age 76.4 years), THC did not reduce NPS (Neuropsychiatric symptoms) compared with placebo; however, it was well tolerated, and the incidence of adverse events was similar between treatment groups [84]. Another study where 24 patients were given THC (oral dose of 4.5 mg/day) and 26 received a placebo found that THC was well tolerated but did not come with a significant reduction in neuropsychiatric symptoms compared to placebo [85]. A randomized trial assessing the safety, pharmacodynamics, and pharmacokinetics of THC involved gradual dosing at 0.75 mg and 1.5 mg in dementia patients over 12 weeks. The findings indicated minor adverse events, with pharmacokinetics showing a dose-linear relationship but notable inter-individual variability [86]. Additionally, a case report documented marked improvements in emotional state, anxiety, and aggressive behaviors in a severe AD patient following dronabinol administration (4.9–6.7 mg/day) [87]. Moreover, THC/CBM-based oil medication in severe dementia patients significantly reduces confusion, agitation/aggression, irritability, apathy, sleep, and caregiver distress [88]. However, further research with larger cohorts is warranted to explore the efficacy and tolerability of elevated THC doses among older adults with dementia. Currently, a randomized controlled trial (NCT05822362) is in progress to investigate the effects of cannabidiol on validated biomarkers of AD progression (e.g., plasma *N*-p-tau181, Aβ42/Aβ40 ratio, Neurofilament Light (NFL)), neurocognitive functions, and clinical outcome measures while also examining potential mechanisms of action. Present clinical evidence does not support the use of cannabinoids as a treatment for cognitive decline in AD. However, it strongly supports their potential use as a safe and effective treatment for managing the behavioral and psychological symptoms of dementia (BPSD), particularly agitation and aggression. This offers a valuable non-antipsychotic alternative for symptom management, which is a significant clinical need.

### 4.2. Curcumin: The Bioavailability Challenge

**Curcumin** is a polyphenolic bioactive curcuminoid extracted from turmeric rhizomes with diverse pharmacological properties [89]. Experimental studies indicate that curcumin can mitigate inflammation and oxidative stress associated with low-density lipoprotein (LDL) while simultaneously improving both overall memory function and non-spatial memory in senescent rodent models exhibiting cognitive deficits [90,91,92,93]. Curcumin is one of the most studied BCs in the spice turmeric to date; however, there is little data on its clinical diagnostic and therapeutic potential in AD human trials. In a preliminary diagnostic study, using curcumin as a fluorescent agent for retinal imaging successfully enabled visualization of Aβ with 80.6% specificity in differentiating between AD and non-AD [94]. Curcumin supplementation (180 mg/day) for 12 weeks significantly reduced circulating GSK-3β and islets APP levels in a high-risk group of T2D and AD [95]. This suggests a potential use of curcumin for reducing the risk of T2D and AD by alleviating insulin resistance-related markers. A solid lipid curcumin formulation, specifically 400 mg of Longvida^®^ (equivalent to approximately 80 mg of curcumin), has been shown to enhance cognitive function and decrease levels of fatigue and psychological stress in a healthy elderly demographic. Additionally, this supplement had a positive effect on both total cholesterol and LDL cholesterol levels [96]. However, in mild cognitive impairment and AD patients (NCT00164749), curcumin’s effect on neurocognitive and biomarker (Serum Aβ40) measurement was not significant, and it did elevate vitamin E levels without any adverse effects at a high dose [97]. In a 24-week placebo-controlled trial (NCT00099710), the efficacy of Curcumin C3 Complex (^®^) in AD could not be established, as the study faced significant subject withdrawals attributable to gastrointestinal discomfort. Consequently, no clinical or biochemical evidence of effectiveness was found. Preliminary results also showed limited bioavailability, with no significant changes in plasma Aβ1-40, Aβ1-42, cerebrospinal fluid (CSF) Aβ1-42, CSF tau, or phosphorylated tau (p-tau) levels in the treatment group [98]. The lack of success in clinical trials may be attributed to the poor bioavailability of curcumin, the selection of cohorts in advanced stages of AD, and disparities in the biology of rodent models and AD patients [99]. One clinical trial combining curcumin and Bioperine was halted (NCT00595582) while another trial utilizing a high-bioavailability curcumin formulation (Longvida) improved aspects of mood and working memory in a healthy older group [100]. Both trials employed formulations designed to enhance curcumin bioavailability. However, despite compelling multi-target preclinical evidence, the absence of clinical efficacy in AD dementia may suggest that bioavailability limitations and the timing of intervention remain critical constraints. These findings support the hypothesis that curcumin’s therapeutic potential may be more relevant to early-stage intervention, prevention strategies, or further advances in formulation technologies.

### 4.3. NIC5-15 (D-Pinitol): A Discontinued Candidate

**NIC5-15** (D-pinitol) is a cyclic sugar alcohol, which acts as an insulin sensitizer and is found in pine bark, soy, etc [101]. In preclinical animal models, NIC5-15 reduces the production of Aβ1-42 by inhibiting notch-sparing γ-secretase [102]. In a phase IIa trial at the VA Medical Center in the Bronx, New York, and the Icahn School of Medicine in Mount Sinai, New York, NIC5-15 was tested in 15 individuals (in doses of 1.5, 3.0, and 5.0 g for up to seven weeks) with mild to moderate AD. Preliminary findings presented at the ICAD conference in Vienna in 2009 suggested good tolerability and potential cognitive stabilization as measured by the ADAS-Cog scale (NCT00470418). Furthermore, this study indicated that NIC5-15 is appropriate for evaluation in an extended feasibility trial [101]. Subsequently, in 2012, a patent was granted for the use of D-pinitol in AD treatment. Following this development, a separate Phase IIb study, involving 40 patients with mild to moderate AD, focused primarily on assessing cognition as measured by ADAS-Cog (NCT01928420). However, the results of this trial have not yet been updated. The clinical evaluation of NIC5-15 (D-pinitol) in the context of AD concluded over a decade ago following a limited Phase II trial that did not demonstrate efficacy. While the compound was deemed safe, it failed to show significant improvements in cognitive function or overall global assessments in patients with mild to moderate AD. Consequently, NIC5-15 serves as a historical case study of a DBs candidate that, despite a plausible mechanism, failed to demonstrate clinical efficacy in early-phase trials and has been rightfully abandoned in the modern therapeutic landscape for AD.

### 4.4. Vitamin E (α-Tocopherol): Symptomatic Benefit Without Cognitive Impact

**Alpha-tocopherol** (Vitamin E) is a lipophilic monophenolic compound that can donate a hydrogen atom to saturate and detoxify the unpaired electron of a free radical. Thus, it is thought to protect brain cells from free radical-induced toxicity. Several epidemiological studies have found that there may be a link between vitamin E supplementation and a reduced risk of developing AD. For example, a prospective study of 633 individuals in 1998 found that none of the 27 vitamin E supplement users developed AD after a follow-up period of 4.3 years [103]. Similarly, a cohort study conducted by Engelhart et al. in 2002 in the Netherlands found similar results at a six-year follow-up [104]. Another study, conducted from 1993 to 2000, suggested that consuming food containing vitamin E (but not other antioxidants) may be associated with a reduced risk of AD, specifically among individuals not carrying the APOE ε4 allele [105]. Additionally, the Cache County Study from Utah, USA, found that individuals supplemented with a multivitamin complex containing vitamin E and vitamin C had a reduced risk of AD; however, no evidence was shown of a protective effect associated with the intake of these compounds alone [106].

The Rotterdam Study involving 365 AD patients found a minor reduction in the long-term risk of AD among participants with a higher intake of vitamin E-rich foods. Conversely, those with average vitamin E intake did not show a lower risk of dementia [107]. Similarly, the Canadian Study of Health and Aging, which included 560 AD patients, suggested that vitamin E supplements were associated with a reduced risk of cognitive decline over the long term [108]. However, other studies have not found a significant association between vitamin E intake and the risk of developing AD. For example, one study following 2969 participants for 5.5 years found that supplemental vitamin E and C did not reduce the risk of AD or overall dementia [109]. Similarly, a study involving 3385 men from the Honolulu–Asia Aging Study suggested that vitamin E and C supplements may improve cognitive function in late life, but did not show a protective effect specifically for Alzheimer’s dementia [110]. Finally, a study of 980 elderly subjects found no association between dietary supplements or total intake of vitamin E and a reduced risk of AD [111]. These disparities in results suggest that further research is needed to fully understand the relationship between vitamin E and the risk of AD. In summary, high-dose vitamin E stands as the sole exception among DBs by demonstrating a significant, albeit modest, slowing of functional decline in AD dementia; however, its lack of cognitive benefit and failure to alter disease course firmly position it as a symptomatic therapy rather than a disease-modifying agent. Despite evidence suggesting modest functional benefits, high-dose vitamin E supplementation remains clinically debated, particularly in light of inconsistent findings and reported dose-related safety concerns [112]. Accordingly, supplementation decisions should consider individual risk–benefit profiles.

### 4.5. Tricaprilin (AC-1202): A Ketogenic Strategy with Subgroup Effects

**Tricaprilin (AC-1202):** Decreased regional cerebral glucose utilization is a characteristic of early-stage AD [113]. AC-1202 is an oral formulation of caprylic triglyceride that improves mitochondrial metabolism by inducing mild chronic ketosis. Tricaprilin is formulated to enable the metabolism of caprylic acid to produce Acetyl-CoA through the formation of ketone bodies. This acetyl-CoA acts as a fuel alternative to glucose to produce energy via the citric acid cycle [114].

In a clinical trial, daily administration of AC-1202 for 90 days in 152 subjects with mild to moderate AD led to a rapid increase in serum ketone bodies in AD patients, improving ADAS-Cog scores. However, the effects were most notable in participants with the APOE4(-) allele. Significantly, further assessment trials concluded that APOE variants may influence the cognitive response to induced ketosis in patients with mild to moderate AD (NCT00142805) [115,116]. In another trial (AC-12-010, Nourish AD), 413 patients with mild to moderate AD stratified by APOE genotype (i.e., APOE4 non-carriers) were randomized for 26 weeks of AC-1204 treatment. Administration of AC-1204 was safe and well-tolerated; however, the study observed no significant difference in cognitive functions (ADAS-Cog) compared to the placebo. Several factors contributed to the lack of efficacy in the study, including lower-than-expected formation of ketone bodies from AC-1204 and a lack of recurrence in patients who received a placebo (NCT01741194) [117]. Critically, the consistent signal in APOE4 non-carriers across multiple trials illustrates a core principle of precision nutrition: genotype can determine whether a metabolic intervention succeeds or fails [118]. Preclinical evidence demonstrates that APOE4 brains may be deficient in ketone body transport and utilization, providing a mechanistic basis for this differential response [119]. The tricaprilin story highlights a mechanistically rational ketogenic strategy whose failure in broad AD populations, coupled with a signal in APOE4 non-carriers, powerfully illustrates the necessity of genotype-stratified enrollment and biomarker-guided participant selection in future trials [120,121]. Subsequent studies should therefore enroll APOE4-stratified populations and incorporate ketone monitoring to confirm target engagement, rather than testing the intervention in unselected AD cohorts.

### 4.6. Docosahexaenoic Acid (DHA): Failed in Dementia, Potential in Prevention

**Docosahexaenoic acid (DHA)** is an abundantly present PUFA (Polyunsaturated fatty acid) in the human brain and is essential for maintaining normal brain function. A higher intake of dietary DHA, enriched in fatty fish, walnuts, and flaxseed, is associated with a lower risk of AD [122,123]. Early clinical trials found a weak or no association between DHA levels in the blood and dementia risk [124,125]. However, recent studies suggest that higher blood levels of DHA or its intake may reduce the risk of cognitive dysfunction or AD [126,127,128]. In AD patients, reduced baseline serum DHA was associated with a higher risk of cognitive impairment [129]. In another cross-sectional and retrospective longitudinal analysis among an elderly population, a lower index of erythrocyte omega-3 was connected with cognitive decline and greater Aβ accretion, whereas, in APOE carriers, it was associated with higher tau aggregation [130].

In the largest multidomain clinical trial of DHA, in older adults (aged 70 with subjective memory loss), 800 mg of DHA had no positive effect on cognitive decline, as measured by composite scores on recall, orientation, processing, and verbal fluency tests. Further sub-studies showed that DHA alone did not affect brain structure and cortical amyloid status [131,132,133,134]. In one study, preliminary results of a daily dose of 1.65 g of EPA plus DHA supplementation on vascular cognitive aging in older adults indicated that supplementation did not slow the accumulation of white matter hyperintensities. However, subsequent analysis showed that participants who received higher DHA and EPA plasma levels had fewer white matter hyperintensities and better white matter integrity than those placebo [135].

A study on the effects of DHA supplementation was carried out at 51 clinical research facilities in the US and involved patients with mild to moderate AD with MMSE scores of 14–16 who were given 2 g of algal DHA per day for 18 months. The analysis indicated that DHA did not have a significant effect on the rate of cognitive and functional decline in AD patients (NCT00440050) [136]. Few other clinical trials examining the effectiveness of DHA in treating AD did not find any significant improvements in cognitive or functional abilities NCT00211159, NCT01058941, NCT01780974, NCT00090402) [137,138,139]. Notably, secondary analyses from multiple trials revealed that treatment response may be modified by genetic background and baseline nutrient status. A systematic review examining interactions between dietary fatty acids and APOE genotype concluded that while APOE4 carriers tend to be most responsive to changes in dietary fat, protective effects of long-chain omega-3 on cognitive decline are observed primarily in non-carriers [140]. In the MAPT study, DHA supplementation showed cognitive benefits only in participants with lower baseline omega-3 indices, while APOE4 carriers exhibited attenuated responses compared to non-carriers [131]. These findings suggest that future DHA trials should stratify participants by APOE genotype and baseline fatty acid status, moving toward a precision nutrition framework rather than assuming uniform efficacy across heterogeneous populations [141]. This pattern provides direct evidence that stratification by APOE genotype changes effect estimates—what appears ineffective in unselected populations may show benefit in genetically defined subgroups. The ongoing PreventE4 trial (NCT03660683) is actively investigating the mechanistic basis for this differential response, testing the hypothesis that APOE4 limits DHA delivery to the brain through altered phospholipase A2 activity [142].

Based on ClinicalTrials.gov data, DHA supplementation shows consistently negative results in improving cognitive function or slowing decline in patients with established AD dementia. However, when evaluated within a precision nutrition framework accounting for APOE genotype and baseline omega-3 status, it may have potential benefits for prevention in cognitively healthy older adults or those with early memory complaints.

### 4.7. Resveratrol: Validated Biomarker Activity Without Clinical Translation

**Resveratrol (RS)** is a polyphenol enriched in grapes, blueberries, peanuts, and plums. It has neuroprotective properties due to its antioxidant and anti-amyloid activity, and it also activates the anti-aging enzyme sirtuin 1. While RS can be included in a healthy diet or taken as a dietary supplement, its ability to reach the brain may limit its potential to treat AD [143,144]. Ongoing clinical studies are investigating RS supplementation for various medical applications, and researchers are also developing small-molecule derivatives with enhanced pharmacokinetic properties.

A phase II trial in the USA assessed the effects of 2 g/day RS capsule supplementation in 119 individuals with mild to moderate AD. The study found that only 1% of the administered RS reached the CSF. Furthermore, whereas the placebo group showed a greater reduction in plasma and CSF Aβ40 levels, the treatment group, in contrast, displayed more significant brain atrophy [145]. In another study, the effects of consuming 1 g of RS orally twice daily for 52 weeks were examined in 119 mild–moderate AD patients by measuring AD-related neuroinflammatory markers in CSF and plasma samples. In this study, RS was found to decrease MMP9 (matrix metalloproteinase-9) levels in CSF (Cerebrospinal fluid), curb neuroinflammation, and persuade adaptive immunity while attenuating declines in MMSE scores [146]. In a recent randomized controlled trial, no significant improvement in verbal memory was found after 6 months of RS supplementation in healthy older adults (aged 60–79 years) with a wide range of Body mass index (BMI). There was, however, a non-significant trend suggesting potential positive effects on pattern recognition memory. The study also noted possible confounding effects due to unfavorable changes in lifestyle behavior, neurotrophins, and inflammatory markers (NCT02621554) [147]. Moreover, 1 g/day of RS supplementation in older adults for 90 days only improved psychomotor speed with no effect on other domains of cognitive function [148]. A recent investigation explored a multifaceted nutritional intervention strategy, comprising omega-3 polyunsaturated fatty acids (PUFAs), vitamin D, resistant starch, and whey protein, to enhance cognitive function in older adults. However, the results indicated only modest efficacy within this demographic. Further research should investigate longer supplement durations or concentrate on populations with specific cognitive deficits to ascertain potential clinical benefits (NCT02001831) [149]. The trials demonstrated definitive evidence of RS’s biological activity within the human Alzheimer’s brain, highlighting its ability to significantly reduce amyloid-beta levels. This significant finding validates the proposed mechanism of action in a human context. However, the lack of efficacy in slowing cognitive decline implies that merely reducing soluble amyloid is insufficient to modify the clinical trajectory of the disease during the mild-to-moderate stages.

### 4.8. Epigallocatechin Gallate (EGCG): Shifting Focus to Early Intervention

**Epigallocatechin Gallate (EGCG)** is a flavonoid in green tea noted for its potential neuroprotective effects through various biological pathways, including gene expression, growth factor signaling, antioxidant activity, and protein degradation. Studies suggest that EGCG may promote the lysosomal degradation of Aβ42 aggregates, enhance α-secretase cleavage of the amyloid precursor protein, and improve cognitive, synaptic, inflammatory, and metabolic phenotypes associated with AD in vivo [150,151,152,153,154]. However, its bioavailability in vivo has been questioned, leading to ongoing efforts to develop synthetic analogs with more potent pharmacological properties [155].

A phase II clinical trial conducted in 2012 evaluated the effects of EGCG on AD-like symptoms, including cognitive outcomes and plasma Aβ biomarkers in 84 individuals (aged 14–29) with Down syndrome. Participants receiving ECGC and cognitive training performed significantly better on memory, executive function, and attention tests than placebo; however, Aβ biomarkers were not reported due to technical issues [156,157]. In January 2014, a Phase III trial with 92 participants began to assess EGCG in multiple-system atrophy (MSA) at Ludwig Maximilians Universität and Technische Universität in Munich. The high-dose EGCG treatment (equivalent to 50 cups of green tea) over one year showed no significant effect on the primary outcome and led to liver damage in some participants, as reported at the April 2020 AAT-AD/PD conference [158]. A recent study (NCT03978052) investigated the effectiveness of a year-long intervention using ECGC to prevent cognitive decline in individuals with the APOE4 gene and subjective cognitive impairment. The study included 200 participants split into four groups, who were given either ECGC or a placebo along with various activities. The maximum ECGC dosage administered was 520 mg/day. The primary focus was to assess cognitive function using a Preclinical Alzheimer’s Cognitive Composite-like battery (PACC) [159]. After 6 months, participants in the multidomain intervention (MC) group showed significant cognitive improvements compared to the lifestyle interventions (CG) group, as measured by the ADCS-PACC-plus-exe (Cohen’s d = 0.38) and the MoCA (Cohen’s d = 0.53). The MC participants also exhibited higher adherence to the Mediterranean diet (76.8 ± 10.9% vs. 60.9 ± 14.9%). This suggests that individualized multidomain lifestyle interventions could be an effective treatment for preventing cognitive decline in those at high risk of developing AD [160]. Currently, there is a notable lack of substantial Phase III clinical trials investigating the efficacy of EGCG in patients with AD dementia. Consequently, the limited clinical data on EGCG epitomize the field’s strategic pivot, with research activity focused solely on early intervention and pre-clinical populations (e.g., Down syndrome), acknowledging that its window of opportunity likely closes well before the onset of dementia.

### 4.9. Nicotinamide Riboside (NR): An Emerging Target for Bioenergetics

**Nicotinamide riboside (NR)** is an analog of nicotinamide and is believed to offer improved bioavailability and pharmacokinetics compared to other forms of vitamin B3 commonly found in various supplements [161]. Cells utilize NR for synthesizing NAD+, a vital coenzyme for many processes, which becomes depleted with aging, affecting processes like Aβ/tau pathology, oxidative stress, mitochondrial activity, ATP production, and DNA repair. Strategies are being explored to enhance NAD+ levels to promote metabolic health, slow aging, and potentially treat age-related diseases [162].

Several Phase I studies have shown that taking up to 1 g/day of NR supplements for eight weeks is safe and well-tolerated in healthy adults. The studies found an increase in blood NAD+ levels and other metabolites starting from week two, which remained elevated for the duration of the study [163,164]. In a study (NCT02942888) on adults with MCI, 20 subjects were given either a placebo or NR with a final dose of 1 g/day over 10 weeks. NR increased NAD+ concentrations by 2.6-fold in blood but did not affect cognition. Although cerebral blood flow (CBF) was reduced after treatment, further trial with an extended period is needed to determine its potential to improve cognition and CBF in older adults with MCI [165]. A randomized clinical trial (NCT04044131) studied the effect of combined metabolic activators (CMAs) containing 1 g of nicotinamide riboside (5%) on AD patients. A notable reduction in the ADAS-Cog score was recorded on day 84 within the CMA cohort, accompanied by enhancements in cognitive function. Additionally, pertinent modifications in hippocampal volumes and cortical thickness were documented. Plasma levels of proteins and metabolites associated with NAD+ and glutathione metabolism were significantly improved after CMA treatment [166]. In a study (NCT02921659) involving 22 healthy older adults, NR supplements (500 mg, twice a day for 6 weeks) led to an upsurge in NAD+ levels in neuronal extracellular vesicles (NEVs) and a diminution in NEV levels of Aβ42, PJNK, and pERK1/2. Alterations in NAD(H) concentrations were found to correlate with fluctuations in insulin-Akt signaling pathways, as well as the activation of PERK1/2 and PJNK. These results suggest that the oral administration of NR has the potential to elevate NAD+ levels in neuronal tissue and influence biomarkers linked to neurodegenerative diseases (ND) in humans [167].

A 2024 systematic review of NAD+ precursors in AD identified two human clinical trials showing marked improvements in plasma biomarkers, neuroimaging measures, and cognitive outcomes following NR supplementation [168]. Most recently, a 2025 randomized controlled trial (NCT04430517) in 46 older adults with subjective cognitive decline and MCI reported that 8 weeks of NR supplementation (1 g/day) significantly lowered plasma pTau217 concentrations compared to placebo (7% reduction vs. 18% increase, *p* = 0.02), although no changes were observed in cognitive performance as measured by the RBANS or other digital assessments [169]. These results demonstrate target engagement at the biomarker level, but the wide inter-individual variability in NAD+ elevation suggests that baseline metabolic status may influence response. A 2023 meta-analysis emphasized that studies to date have not separated NR responses based on gender, age, BMI, or health status, preventing personalization of dosing recommendations. The authors recommended that future studies include heterogeneous response analyses to identify responders and non-responders [170]. An ongoing dose-optimization trial (NCT05617508, N-DOSE AD) is currently investigating the optimal biological dose of NR (1000–3000 mg/day) required to achieve maximal cerebral NAD+ increase in AD patients, with expected completion in 2025.

Currently, nicotinamide riboside is undergoing active clinical investigation, including a Phase II trial at McLean Hospital (NCT04430517) examining effects on brain energy metabolism, oxidative stress, and cognition in MCI and mild AD, with primary completion expected in 2025. Based on the completed and ongoing trials registered on ClinicalTrials.gov, the clinical investigation of NR for AD and cognitive decline is advancing from early-stage safety studies toward biomarker-defined efficacy trials. When interpreted through a precision nutrition lens, NR remains a promising candidate for metabolically defined subgroups, though it has yet to be established as an effective treatment or preventive measure for unselected populations.

## 5. Specific Translational Gaps for Nutritional Interventions in AD

The largely negative outcomes observed in the high-dose DBs trial conducted in dementia-stage populations, despite promising preclinical findings, point to several translational challenges. Unlike many infectious diseases, where a single causative agent can be targeted, AD involves a complex, multifactorial pathophysiology that unfolds over decades. Critically, significant and irreversible neuronal loss precedes the onset of clinical symptoms. By the time dementia is diagnosed, therapeutic strategies are largely confined to symptom management or slowing progression, as rescuing extensively damaged neurons is exceedingly difficult. This late-stage intervention paradigm is a fundamental barrier not only for pharmaceuticals but also for high-dose DBs trials [171].

The development of disease-modifying therapies for AD has been marked by a high failure rate, with over 99% of clinical trials failing to differentiate from placebo. To date, only symptomatic treatments (cholinesterase inhibitors and an NMDA receptor antagonist) are approved. While the focus has shifted toward combination therapies targeting multiple pathways, a strategy that aligns well with the pleiotropic nature of dietary bioactives, no such therapy has yet succeeded in Phase III trials [172]. This highlights a critical gap: the lack of validated therapeutic targets and the inherent difficulty of intervening effectively in a complex, late-stage disease process. Moreover, this landscape of multiple, partially validated targets, encompassing amyloid, tau, APOE-4 biology, neuroinflammation, and mitochondrial function, presents both a challenge and an opportunity for DB development. Unlike single-target pharmaceuticals, many dietary bioactives inherently modulate multiple pathways simultaneously, aligning with the multifactorial nature of AD [173].

**Figure 5:**
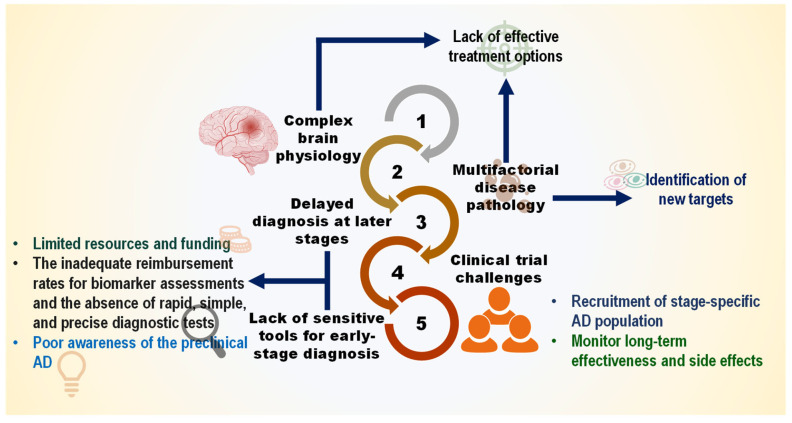
Key Challenges in AD breakthrough.

A primary obstacle in understanding AD etiology is the disconnect between the initiation of pathology and clinical manifestation. Identifying individuals in the pre-symptomatic or prodromal stages is essential for testing preventive interventions. Advances in blood-based biomarkers (e.g., phosphorylated tau, Aβ42/40 ratio) now offer the potential to detect AD pathology 15–20 years before dementia, creating a crucial window for preventive nutritional trials [171,174]. These advancements, however, are coupled with persistent challenges in trial design and implementation (Figure 5). Furthermore, uncertainty remains about the causative role of hallmark proteins, like Aβ and tau, and whether targeting them alone is sufficient [175]. For nutritional and nutraceutical research to realize its preventive potential, the following specific translational hurdles must be decisively addressed:

### 5.1. The Bioavailability-Bioactivity Disconnect

A primary translational hurdle for dietary bioactives in AD is the profound disconnect between in vitro neuroprotective efficacy and in vivo bioavailability. Promising compounds such as curcumin and resveratrol exhibit potent, multi-target effects in cellular and animal models, often at micromolar (µM) concentrations. However, their clinical translation is severely hampered by extremely low systemic bioavailability, rapid metabolism, and poor blood–brain barrier (BBB) permeability in humans, resulting in brain concentrations that are frequently orders of magnitude lower (nanomolar, nM range) than those required for efficacy in preclinical studies [176]. This gap renders compelling in vitro data largely irrelevant unless the compound can be reliably delivered to its intended site of action in the human brain.

The challenge of BBB penetration is not unique to DBs; it has been a critical determinant of failure in pharmaceutical development for AD [177]. The human BBB, with its efflux transporters and selective permeability, is imperfectly modeled in rodents [178]. Consequently, demonstrating neuroprotection in animal models is insufficient without concurrent proof of central target engagement in human trials. A rigorous translational framework for DBs must therefore adopt principles from drug development: prioritizing advanced food-grade delivery systems (e.g., lipid nanoparticles, phospholipid complexes, cyclodextrin inclusion) to enhance stability and brain uptake [176], and incorporating phase I pharmacokinetic studies that measure cerebrospinal fluid (CSF) concentrations to estimate brain exposure [179].

Ultimately, for a dietary bioactive to be considered a viable candidate, clinical trials must move beyond simply reporting cognitive outcomes. They must pair these endpoints with validated biomarkers confirming that the compound reaches the brain (pharmacokinetics) and successfully modulates its intended pathological target (pharmacodynamics) [173]. Without closing this bioavailability-bioactivity loop, clinical trials of poorly bioavailable DBs are unlikely to succeed, regardless of the strength of their preclinical rationale. However, even when adequate bioavailability and central target engagement are achieved, as demonstrated by resveratrol’s reduction in CSF Aβ40, clinical benefit does not automatically follow. This raises a second critical question: how should biomarker evidence be interpreted? The following subsection provides a framework for distinguishing target engagement from true surrogate endpoints.

### 5.2. Interpreting Biomarker Outcomes: Target Engagement vs. Surrogate Endpoints

The dissociation between biomarker effects and clinical outcomes observed with several dietary bioactives, particularly resveratrol-associated reductions in CSF Aβ40 without measurable cognitive benefit, underscores a critical distinction in AD trial interpretation: the difference between target engagement biomarkers and validated surrogate endpoints [180,181].

Target engagement biomarkers provide evidence that an intervention modulates its intended biological pathway, thereby confirming pharmacodynamic activity rather than clinical efficacy. In the case of resveratrol, reductions in CSF Aβ40 suggest central pharmacodynamic effects consistent with modulation of amyloid-related processes [145]. Similarly, nicotinamide riboside (NR) increases systemic NAD+ availability and has been associated with alterations in metabolic pathways relevant to neuronal bioenergetics [167]. Such findings establish that these compounds exert measurable biological effects in humans, directly addressing the bioavailability-bioactivity gap discussed in Section 5.1.

However, target engagement does not inherently imply clinical benefit. For a biomarker to function as a surrogate endpoint, two additional conditions must be satisfied: (i) the biomarker must be causally linked to the clinical outcome of interest, and (ii) treatment-induced changes in the biomarker must reliably predict clinical outcomes across multiple studies, interventions, and populations [181]. In AD research, amyloid PET reduction has been considered a *reasonably likely surrogate endpoint* in specific therapeutic contexts, whereas many other biomarkers, including CSF Aβ40, remain mechanistically informative but are not clinically validated predictors of cognitive outcomes [182,183].

Importantly, the lack of validation of most biomarkers as clinical surrogates significantly limits the interpretation of positive mechanistic signals. Therefore, although reductions in CSF Aβ40 or increases in NAD+ demonstrate target engagement, these effects should be interpreted as preliminary mechanistic evidence rather than proof of therapeutic efficacy. This perspective reinforces the rationale for early-stage trials in biomarker-defined populations, where disease mechanisms remain active and potentially modifiable, as further developed in Section 5.3.

### 5.3. Lack of Biomarker-Driven Personalized Nutrition

The interpretive framework above highlights that even when a compound engages its target, individual variability in response can obscure efficacy signals. Response to nutritional interventions is highly heterogeneous, influenced by genetics (e.g., APOE status), gut microbiome composition, metabolic health, and baseline nutrient status [184]. This biological variability presents a critical translational gap: the current “one-size-fits-all” approach to nutritional trials fails to account for individual differences that fundamentally determine therapeutic efficacy. As a result, interventions that might benefit specific subgroups appear ineffective when tested in unstratified, heterogeneous populations.

Emerging evidence from DHA and tricaprilin trials demonstrates that genotype stratification can reveal treatment effects otherwise obscured in heterogeneous populations. For DHA, systematic reviews confirm that APOE4 status consistently modifies response to omega-3 supplementation, with cognitive benefits observed primarily in non-carriers [140]. For tricaprilin, multiple trials showed cognitive improvements only in APOE4-negative participants [118,120,121]. These findings provide empirical validation that precision nutrition approaches can change effect estimates in AD nutritional interventions.

However, for most dietary bioactives, including nicotinamide riboside, curcumin, resveratrol, and EGCG, biomarker-based stratification remains a proposed future direction requiring validation in prospective trials. A 2023 meta-analysis of NR studies explicitly noted that trials have not separated responses based on age, BMI, or health status, preventing personalization of dosing recommendations [170]. Similarly, emerging study protocols are now designing personalized dietary supplements for AD patients based on individual gut microbiota profiles (NCT06199193), integrating clinical, dietary, and microbial data using AI and network analysis to identify modifiable biomarkers [185]. While these approaches are still in early stages, they represent the next generation of precision nutrition strategies.

A major gap is the lack of frameworks for “precision nutrition” in AD prevention. Future trials need to move beyond generic designs by strategically stratifying participants using baseline biomarkers to identify “responder” subgroups and understand mechanism-based efficacy [186]. For instance, trials investigating omega-3 fatty acids could pre-select participants based on *APOE* genotype or baseline erythrocyte DHA levels [187], while studies on probiotics or polyphenols might stratify by gut microbiome profiles or inflammatory markers [188].

The ongoing development of sensitive biomarkers, from genetic risk profiles to blood-based indicators of AD pathology (e.g., plasma p-tau, Aβ42/40 ratio), now makes such precision approaches feasible [174]. Implementing this stratified methodology is essential not only to demonstrate efficacy in responsive populations but also to develop truly personalized dietary recommendations for cognitive health preservation [184,186].

### 5.4. The “Supplement vs. Food Matrix” Dilemma

A fundamental translational question in nutritional neuroscience is whether isolated, high-dose DBs can replicate the neuroprotective benefits conferred by whole foods and complex dietary patterns. Most clinical trials to date have employed a reductionist model, testing single bioactive compounds (e.g., curcumin, resveratrol) at supraphysiological doses. This approach risks overlooking the synergistic and additive interactions that occur within a whole food matrix, where fibers, fats, vitamins, and a spectrum of polyphenols co-exist and can enhance bioavailability, stability, and biological activity [189]. For example, the absorption of curcumin is significantly potentiated by piperine in black pepper, and the health effects of olive oil are attributed to the combined action of oleic acid, polyphenols, and vitamin E.

Epidemiological studies highlight that complex dietary patterns, such as the Mediterranean or MIND diets, are associated with a reduced risk of cognitive decline and AD [190]. While these findings cannot be directly extrapolated to isolated DBs, they underscore critical translational considerations for trial design. Specifically, such diets suggest that multi-component, synergistic effects may drive observed benefits, emphasizing the importance of accounting for dietary background, nutrient interactions, and adherence in DBs trials. Translationally, this supports developing trial methodologies that: (1) accurately assess participants’ habitual diet and adherence; (2) control for confounding background nutrient intake; and (3) incorporate biomarkers reflecting systemic or central exposure to multiple bioactives, thereby bridging the gap between isolated compound testing and real-world dietary complexity [191]. Bridging this gap is essential to determine whether the future of nutritional prevention lies in precision DBs, whole-diet recommendations, or a synergistic combination of both.

Overcoming these gaps will require the establishment of a dedicated framework for nutritional translational science. This includes **(1) adopting** target product profiles for DBs, **(2) conducting** rigorous pharmacokinetic/pharmacodynamic studies even for food-derived compounds, and **(3) developing** innovative, cost-effective trial designs that can evaluate long-term, subtle benefits of dietary interventions. By learning from the failures of pharmaceutical development and embracing the unique challenges of nutrition research, the field can bridge the gap between promising preclinical data on dietary bioactives and their meaningful impact on brain health.

## 6. Conclusions and Future Directions: Towards a Preventive Nutritional Strategy for Brain Health

Collectively, the available clinical trial evidence suggests that current approaches to evaluating dietary bioactives in AD may benefit from reconsideration. While recent discoveries have illuminated various factors in sporadic AD, a comprehensive understanding of their intricate interactions remains a frontier. The “multifactorial hypothesis” posits that concurrently targeting multiple pathological pathways could represent a promising therapeutic strategy. This framework may be particularly relevant for nutraceutical research as it explains the limited success of single-target pharmacological agents and aligns with the inherent pleiotropic nature of many dietary bioactives.

Over recent decades, clinical drug development for AD has experienced a high attrition rate, with roughly 90% of candidates failing in trials. Contributing factors include limited clinical efficacy, unacceptable toxicity, suboptimal pharmacokinetics, and strategic challenges [192]. Despite various efforts, the success rate has remained stubbornly low (10–15%). Consequently, there is increasing research interest in DBs for their potential neuroprotective properties, reflected in the increasing number of these compounds entering clinical trials for AD. They are increasingly explored not merely as alternatives to synthetic drugs, but as potential components of a different paradigm, one centered on multi-target modulation, preventive nutrition, and enhanced safety profiles.

This study provides a critical examination of clinical trials investigating dietary DBs and nutraceuticals for the treatment of AD. The publicly available evidence base remains limited, with several trials concluded prematurely. While these compounds are generally well-tolerated, robust evidence for neuroprotective efficacy in patients with established dementia is currently lacking. Most interventions have been assessed only in small-scale or Phase II trials that have not progressed to definitive Phase III studies, often halted due to insufficient efficacy, significant bioavailability challenges, or strategic discontinuation. The inconsistency in cognitive outcomes further underscores the necessity for longer-term, well-powered studies in appropriately defined populations. Importantly, many available trials remain limited by small sample sizes, short intervention durations, and heterogeneous outcome measures, which complicate the interpretation of efficacy.

The available clinical trial evidence does not consistently demonstrate that DBs slow cognitive decline in established AD dementia. These findings are consistent with the widely discussed interpretation that interventions initiated after substantial neuronal damage and clinical symptom onset may be less likely to produce measurable cognitive benefits (“too little, too late”). A partial exception is high-dose vitamin E (α-tocopherol), which demonstrated a significant delay in functional, but not cognitive, decline, suggesting a role in symptomatic or functional management rather than *disease-modifying therapy*. Importantly, interventions at pharmacological doses, including vitamin E, are **not inherently safety-neutral** and require careful clinical monitoring to balance potential benefits with risks such as bleeding or cardiovascular events [193].

**Figure 6:**
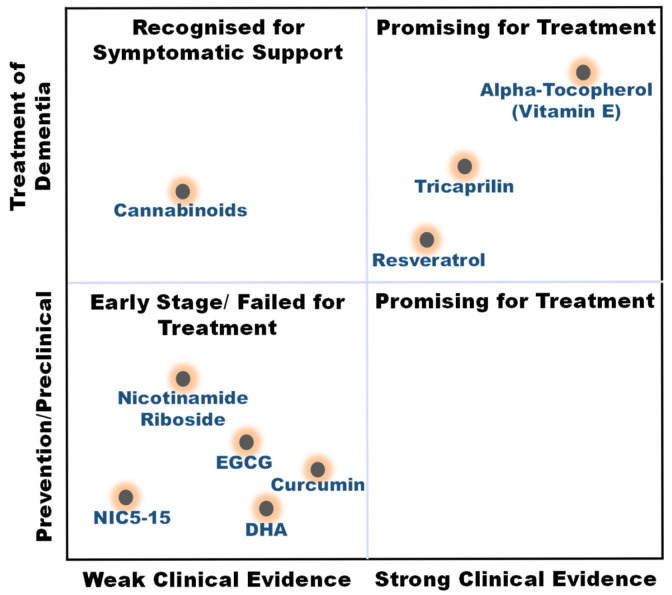
Conceptual summary of the clinical trajectories of dietary bioactives (DBs) evaluated in AD. The diagram illustrates the relative positioning of compounds based on the strength and nature of available clinical evidence, including trial phase, primary endpoint outcomes (cognitive vs. functional vs. biomarker), and development status. Alpha-tocopherol (vitamin E) occupies the upper-right quadrant, reflecting evidence from late-phase trials demonstrating a statistically significant effect on functional decline, although without consistent cognitive benefit. Compounds grouped in the upper-left quadrant represent agents with evidence of biomarker modulation or subgroup-specific signals but without reproducible success on primary cognitive endpoints. For example, tricaprilin demonstrated effects in genotype-defined subgroups, while resveratrol showed measurable biomarker changes. Cannabinoids primarily exhibited effects on behavioral outcomes. Compounds positioned in the lower-right quadrant correspond to agents whose clinical investigations have increasingly emphasized early-stage or preventive contexts following limited efficacy in AD dementia trials. Nicotinamide riboside is included here due to ongoing evaluation in mild cognitive impairment populations. Agents in the lower-left quadrant reflect compounds with discontinued development following unsuccessful clinical trials. This figure represents a qualitative conceptual framework rather than a formal evidence grading system.

The strategic landscape for DBs in AD is visually represented in Figure 6, which depicts nine compounds based on their clinical evidence and application. This quadrant chart illustrates a potential shift in research focus from treating dementia toward preventive strategies, with most DBs now classified as “*Promising for Prevention*.” Despite compelling preclinical evidence, repeated clinical failures likely reflect common challenges: limited bioavailability, suboptimal trial design, and, most importantly, interventions initiated late in the disease course, often after irreversible neuronal damage. Table 1 details how clinical outcomes have diverged, steering future research toward long-term preventive strategies in high-risk, preclinical populations.

For precision nutrition, some evidence suggests that stratification may reveal treatment effects in specific subgroups: DHA and tricaprilin demonstrate cognitive benefits in genotype-defined subgroups (e.g., APOE4-negative), illustrating that targeted interventions can change effect estimates in heterogeneous populations [118,120,121,140]. Future trials may consider genotype-stratified designs, although these approaches remain exploratory until validated in larger, prospective studies.

For DBs, including curcumin, resveratrol, EGCG, and nicotinamide riboside, biomarker-based stratification represents a proposed future direction, requiring prospective trials to identify metabolically or genetically defined responder subgroups (e.g., baseline NAD+ levels, inflammatory status, gut microbiome composition) [170,185]. These compounds represent potential candidates for evaluation in large-scale trials targeting individuals defined by genetic (e.g., APOE4 carriers) or biomarker (e.g., amyloid positivity) risk, intending to address early pathologies such as oxidative stress and neuroinflammation before irreversible neuronal damage occurs. It should be noted, however, that most mechanistic biomarkers remain unvalidated as clinical surrogates, limiting the interpretation of positive target engagement signals as indicators of clinical efficacy.

Collectively, this analysis argues against a high-dose, single-DBs ‘silver bullet’ for AD dementia. Instead, it suggests that future research may increasingly focus on preventive nutritional neuroscience, emphasizing multi-target modulation, early intervention, and biomarker-guided strategies. DBs may confer potential benefits when integrated into brain-healthy dietary patterns, possibly enhanced by formulated DBs for at-risk individuals. While DBs hold promise for preventive strategies, high-dose interventions should be approached cautiously, as pharmacological doses may pose safety concerns. Future clinical trials must integrate risk–benefit assessments and monitor adverse events to ensure safe application. Addressing bioavailability issues with advanced delivery systems (e.g., nanoparticles for Curcumin, EGCG) and verifying target engagement (e.g., CSF NAD+ for NR) remain critical. Since AD has multiple causes, combination approaches targeting multiple pathways (e.g., metabolic agents, anti-inflammatories, antioxidants) warrant exploration in well-designed studies, alongside innovative trial designs and endpoints. Progress in this domain will likely require interdisciplinary approaches integrating nutritional science, formulation technology, and biomarker-guided strategies to generate evidence-informed dietary interventions that may preserve cognitive function and prevent decline across the lifespan.

## Figures and Tables

**Table 1:** The clinical trial summary for the nine dietary bioactives (DBs).

Compound	Mechanism of Action	Phase II Result	Phase III Result	Primary Result	Current Status & Future Direction	Precision Nutrition Status
Alpha-Tocopherol (Vitamin E)	Antioxidant; protects neurons from oxidative damage.	Functional benefits reported	Slower functional decline (ADCS-ADL) observed; no cognitive benefits	Positive: Slowed functional decline by 19% in mild–moderate AD. No cognitive benefit.	In Use. Considered for symptomatic functional support in clinical guidelines.	Not established
Tricaprilin (AC-1202)	Ketogenic substrate; alternative brain energy source.	Failed Phase III primary endpoint; consistent benefit in APOE4 non-carriers only	Primary endpoints not met	Failed primary cognitive endpoints. Signal in APOE4- subgroup.	Stalled. Future studies may require biomarker/genotype stratification.	Evidence-based: APOE4 stratification changes effect estimates
Resveratrol	Activates sirtuins; reduces amyloid-beta levels; anti-inflammatory.	Biomarker changes reported	N/A (No Phase III trials)	Failed primary cognitive endpoints. Positive: Significantly reduced Aβ40 in plasma/CSF.	Research Tool. Used to study amyloid clearance. potential role in mechanistic or combination studies.	Proposed (biomarker stratification not yet tested)
Cannabinoids	Modulates endocannabinoid system; anxiolytic; reduces agitation.	Data heterogeneous/limited	N/A	Pending. Investigated mainly for behavioral and neuropsychiatric symptoms rather than cognition	Experimental for BPSD. Research focused solely on managing behavioral symptoms like agitation and aggression; ongoing biomarker trials (NCT05822362)	Not established
DHA (Docosahexaenoic acid)	Supports neuronal membrane health; anti-inflammatory.	Mixed findings	Primary cognitive endpoints not met	Negative: No reproducible cognitive or functional benefit in established AD.	Prevention Hypothesis. Research shifted to primary prevention or pre-clinical stages.	Evidence-based: APOE4 and baseline omega-3 index modify response
Curcumin	Anti-inflammatory; anti-amyloid aggregation; antioxidant.	Limited or inconsistent findings	Primary endpoints not met	Negative: No cognitive or biomarker benefit in AD. Potential behavioral benefit.	Prevention Hypothesis. Focus on bioavailable formulations for pre-clinical populations.	Proposed (bioavailability may limit stratification utility)
EGCG (Epigallocatechin gallate)	Antioxidant; modulates amyloid precursor protein processing.	Cognitive signals in high-risk groups (Down syndrome trial)	N/A	Positive: Improved visual memory & inhibition in a high-risk population.	Prevention. Promising for pre-clinical/at-risk groups (e.g., Down syndrome, MCI).	Proposed (under investigation in APOE4-stratified trial NCT03978052)
Nicotinamide Riboside (NR)	Boosts NAD+; improves mitochondrial function & DNA repair.	Target engagement under investigation	N/A	Pending. Studies primarily assess biomarker and metabolic outcomes (CSF NAD+ levels); target engagement and cognition.	Ongoing Research. Awaits results. Potential for early intervention in MCI.	Emerging evidence: Target engagement confirmed; stratification by baseline NAD+ or metabolic status proposed but unvalidated
NIC5-15 (D-Pinitol)	Proposed insulin sensitizer; aimed to address metabolic deficits in brain.	No consistent benefit observed: Phase IIa suggested stabilization; Phase IIb results unreported; abandoned	N/A	Negative: No significant effect on cognition or function in available trials	Discontinued. Development halted after failed Phase II trial.	Not applicable (N/A)

## Data Availability

No new data were created or analyzed in this study.
